# Misoprostol in Refractory Neuralgic Pain: From Clinical Neurology to an EP-Receptor Hypothesis of Pharmacodynamic Persistence

**DOI:** 10.3390/ijms27198455

**Published:** 2026-09-22

**Authors:** Ronald B. Bukowy, Dario Rusciano, Caterina Gagliano, Mario D. Toro

**Affiliations:** 1 Independent Researcher, Geneva, IL 60134, USA; bukowyron@gmail.com; 2 Neurovisual Science Technology, Via Tomaselli 43, 95125 Catania, Italy; 3 Faculty of Medicine and Surgery, University of Enna “Kore”, 94100 Enna, Italy; caterina.gagliano@unikore.it; 4 Mediterranean Foundation G.B. Morgagni, Ophthalmology Clinic, 95125 Catania, Italy; 5 Eye Clinic, Department of Public Health, University of Naples Federico II, 80131 Naples, Italy; mariodamiano.toro@unina.it; 6 Department of General and Paediatric Ophthalmology, Medical University of Lublin, 20-079 Lublin, Poland; 7 Department of Special Surgery, University of Jordan, Queen Rania St., Amman 11942, Jordan

**Keywords:** misoprostol, neuropathic pain, trigeminal neuralgia, prostaglandin E receptors, EP receptors, neuroimmune signaling, pharmacodynamic persistence, drug repurposing, corneal pain

## Abstract

This hypothesis originated from the first author’s personal experience in adult neurology, in which selected refractory cranial or postherpetic neuralgias occasionally appeared to improve rapidly after off-label misoprostol. The original records are unavailable; these physician recollections are not efficacy data and serve only as the hypothesis generator. Independent published precedents are limited to small uncontrolled reports in multiple-sclerosis-associated trigeminal neuralgia. A further recollection was that perceived benefit often waned after several weeks at conventional daily exposure, whereas lower or intermittent exposure sometimes appeared to remain useful for longer; this observation is likewise unverifiable and does not define a therapeutic dose. Misoprostol is rapidly converted to misoprostol acid (plasma half-life of 20–40 min). We ask whether a brief, reversible exposure could produce pharmacodynamic persistence, defined here as a reproducible pain-relevant effect that outlasts measurable drug exposure, through EP-receptor trafficking, compartmentalized GPCR signaling, or downstream cellular changes. The model also predicts that the concentration–response relationship may be non-monotonic: increasing or repeated exposure could attenuate or reverse a favorable effect by recruiting opposing EP pathways or adaptive receptor/cellular responses. No direct evidence shows that misoprostol produces such persistence in neural tissue, and PGE2/EP signaling can also be pronociceptive. Receptor-resolved concentration–response and pulse-washout experiments must therefore separately demonstrate receptor signaling, persistent cellular change, altered neuronal excitability, and ultimately blinded analgesic benefit. Neuropathic corneal pain is considered only a secondary mechanistic model. No evidence-based dose can currently be recommended.

## 1. Clinical Origin and Rationale

Trigeminal neuralgia (TN) can be profoundly disabling, and carbamazepine or oxcarbazepine remains first-line therapy; intolerance, incomplete response, secondary disease, and recurrence after intervention leave a clinically important refractory group [1]. Although TN is uncommon, its epidemiological burden is not trivial: a recent global synthesis estimated a pooled incidence of 25.33 cases per 100,000 person-years while emphasizing wide between-study heterogeneity related in part to case definition and ascertainment [2]. At the molecular level, chronic neuropathic pain is not simply prolonged acute nociception: peripheral excitability, synaptic plasticity, glial activation, immune-cell recruitment, and altered signaling at the injured nerve can sustain a pathological state after the initiating lesion has changed or resolved [3,4,5].

The present hypothesis did not begin with a literature search. It arose from the personal clinical experience of the first author, Ronald B. Bukowy (R.B.B.), during several decades of adult neurological practice. That origin is retained because it explains the question being asked, but the recollections are deliberately separated from the evidentiary basis of the article. The verifiable published literature is therefore summarized first, before the historical observation is described in detail.

Independent of R.B.B.’s recollections, a small published clinical literature provides the only direct human precedent. In 1995, Reder and Arnason described seven patients with multiple-sclerosis-associated trigeminal neuralgia refractory to conventional treatment; six were reported to improve with misoprostol [6]. An open prospective study subsequently enrolled patients with the same secondary neuralgia: 18 completed treatment with 600 μg/day and 14 showed reductions greater than 50% in both attack frequency and pain intensity, beginning approximately five days after treatment onset [7]. A 2012 letter reported three additional therapy-resistant cases [8]. These reports are summarized structurally in Section 6. None was a modern randomized, placebo-controlled efficacy trial, and none provides evidence for a 60–72 h post-dose analgesic state.

R.B.B. recalls prescribing off-label misoprostol to patients he personally treated for refractory trigeminal, glossopharyngeal, lesion-associated cranial, or postherpetic neuralgic pain. Some appeared to improve rapidly; others did not respond, developed gastrointestinal intolerance, or experienced waning benefit. In his recollection, sustained treatment at approximately conventional daily doses, often around 600 μg/day, frequently lost perceived efficacy after roughly 4–6 weeks, whereas lower or intermittent exposure sometimes appeared to retain benefit for substantially longer. One memorable patient reportedly used 100 μg only when the first pain ‘twinge’ returned, commonly after about 60–72 h, and this pattern was recalled as remaining useful over a prolonged period of follow-up. The original records are no longer available, the observations arose in routine care rather than a research protocol, and no denominator, standardized endpoint, or patient-level reconstruction is possible. Because redosing was triggered by pain recurrence, the interval is intrinsically coupled to the dosing decision and is not an independent measure of drug effect. Likewise, the remembered contrast between conventional and lower exposure cannot establish a dose–response relationship, dose optimum, or duration of efficacy. These physician recollections are therefore not efficacy data, a case report, or a retrospective case series; they function solely as the clinical heuristic that generated two falsifiable questions: whether a brief exposure can produce a post-exposure effect, and whether the relevant exposure–response relationship is non-monotonic rather than simply dose-proportional.

This hypothesis article tests that clinical question against current molecular pharmacology. We distinguish throughout among (i) evidence directly involving misoprostol or misoprostol acid, (ii) indirect evidence from PGE2 or other prostanoid ligands, and (iii) general GPCR mechanisms derived from unrelated receptors. The central question is not whether the historical observation was ‘true’, but whether a reproducible post-exposure effect can be demonstrated prospectively and linked, step by step, to EP-receptor engagement, a persistent cellular alteration, altered neuronal excitability, and finally analgesia.

## 2. Hypothesis Development and Literature Approach

To develop and challenge the hypothesis, PubMed/MEDLINE and the reference lists of retrieved publications were examined from database inception through 9 September 2026. Search combinations paired misoprostol or misoprostol acid with trigeminal neuralgia, postherpetic neuralgia, neuropathic pain, EP1, EP2, EP3, EP4, receptor binding, receptor affinity, receptor trafficking, internalization, GPCR endosomal signaling, mast cells, macrophages, microglia, astrocytes, pharmacokinetics, tissue exposure, corneal pain, ocular delivery, safety, and drug availability. Searches were iteratively refined when a retrieved study identified a receptor subtype, pharmacokinetic issue, or competing explanation requiring targeted follow-up.

Priority was given to primary clinical reports, official prescribing information, receptor-binding and structural studies, mechanistic human-cell work, and in vivo models with interpretable receptor biology. English-language publications were prioritized; human, animal, and in vitro studies were retained when they directly informed the clinical signal, receptor mechanism, temporal hypothesis, competing explanations, ocular translation, or safety. Titles and abstracts were screened for mechanistic relevance, and full text was examined when needed to verify a specific claim. Tangential prostanoid literature without a clear bearing on these questions was excluded. No formal Embase, Scopus, or Cochrane search was performed, which is an explicit limitation. This targeted hypothesis-development process was not designed to estimate treatment effect and should not be interpreted as a systematic or comprehensive review.

Generative artificial intelligence (ChatGPT, GPT-5.6 Sol, OpenAI, San Francisco, CA, USA) was used to support literature organization, manuscript restructuring, language editing, and preparation of conceptual schematic figures. It was not treated as an evidentiary source and did not determine scientific inclusion or exclusion decisions, interpretation of individual studies, or the conclusions of the article. The cited literature and the claims attributed to it were independently checked by the authors. Additional disclosure is provided in the Acknowledgments.

## 3. Molecular Pharmacology of Misoprostol and EP Receptors

Misoprostol is the orally administered methyl-ester parent compound and is rapidly de-esterified to misoprostol acid, the principal circulating pharmacologically active species [9,10,11]. Misoprostol/misoprostol acid should not be used interchangeably with PGE1 (including alprostadil) or with endogenous PGE2; these molecules differ in receptor pharmacology, exposure, and biological context. In healthy adults, plasma misoprostol acid peaks within minutes and has a terminal half-life of approximately 20–40 min [10,11]. The current label reports marked inter- and intra-study variability, dose-proportional mean exposure over 200–400 μg, and no accumulation during repeated dosing [10]. Importantly, plasma half-life is not equivalent to tissue residence time. Neural, ganglionic, or corneal tissue concentrations after oral dosing have not been established, and the label describes further metabolism to prostaglandin-F analogs without evidence that a long-lived metabolite explains neuralgic pain. Thus, prolonged tissue exposure or an unrecognized active compartment cannot be excluded merely from plasma clearance; they must be measured directly.

### 3.1. EP1–EP4 Signaling Is Not a Single Pathway

Prostaglandin E2 acts through four E-type prostanoid receptors (EP1–EP4), all members of the GPCR superfamily [12,13]. EP1 is mainly linked to calcium mobilization; EP2 and EP4 usually stimulate adenylyl cyclase and cyclic adenosine monophosphate (cAMP); common EP3 isoforms inhibit adenylyl cyclase through Gi, although EP3 splice variants diversify coupling [12,13]. These canonical assignments are useful but incomplete. Receptor abundance, ligand concentration, cell lineage, inflammatory state, receptor heteromers or splice variants, and downstream signaling competence can change the net biological output.

Misoprostol acid is not a clean subtype-selective ligand. In recombinant human receptors, reported binding affinities are approximately 7.9 nM at EP3, 23 nM at EP4, 34 nM at EP2, and greater than 10 μM at EP1 [14]. This profile makes substantial direct EP1 engagement at low exposure less plausible than EP2/EP3/EP4 engagement, although binding affinity is not a functional EC50 and cannot be translated directly into tissue occupancy. Structural analysis of misoprostol acid bound to EP3 shows conventional non-covalent ligand recognition [15], while a ligand-bound EP4-Gs cryo-EM structure provides subtype-specific context for orthosteric prostanoid recognition and coupling [16]. These data are direct receptor-level evidence; they do not establish prolonged EP signaling after misoprostol clearance.

Whether the recalled 100 μg oral dose could reach a pain-relevant receptor concentration is unknown. The prescribing information characterizes pharmacokinetics primarily over the 200–400 μg range and reports low pg/mL plasma concentrations with substantial variability [10]; therefore, exposure at 100 μg should not be inferred by simple proportional extrapolation. Conversely, recombinant Ki values [14] cannot be equated with free drug concentration in a nerve, ganglion, or ocular compartment. A pharmacologically credible experimental program should first measure unbound misoprostol-acid exposure and then bracket the EP2/EP3/EP4 affinity range (roughly low-nanomolar to low-hundreds-of-nanomolar concentrations) while clearly separating clinically plausible from supraphysiological conditions.

### 3.2. Desensitization, Trafficking, and Signaling Location

EP2 and EP4 both couple to Gs, yet they differ in regulation. EP4 undergoes rapid agonist-induced desensitization and internalization, whereas EP2 is relatively resistant to short-term desensitization under comparable experimental conditions [17,18]. Human EP4 internalization involves beta-arrestin- and dynamin-dependent mechanisms [18]. In sensory neurons, however, prostaglandin E2 can increase EP4 availability at the cell surface and enhance subsequent responsiveness [19]. The same receptor family can therefore support attenuation or sensitization depending on cell type and sequence of stimulation.

More broadly, GPCR signaling is no longer understood as a plasma-membrane-only event. Internalized receptors can continue or reshape signaling from endosomes and other intracellular compartments [20,21]. Recent biosensor work further supports temporally structured G-protein regulation from endosomal GPCRs [22]. These studies do not demonstrate persistent EP signaling after misoprostol in neuralgia; they provide a molecular vocabulary for testing whether receptor location and trafficking could separate a brief ligand pulse from a longer downstream response.

### 3.3. Pain Biology Predicts Opposing EP Effects

The pain literature warns against assigning fixed analgesic or pronociceptive labels to individual EP pathways. Selective EP3 activation was antinociceptive in one inflammatory model [23]. By contrast, Minami et al. found that PGE2-induced allodynia was abolished in EP1-deficient mice but preserved in EP3-deficient mice, identifying EP1 rather than EP3 as necessary in that experimental paradigm [24]. Misoprostol itself has shown a non-monotonic effect on mast-cell histamine release [25]. EP2 and EP4 signal through Gs/cAMP; EP4 can support sensory-neuron sensitization, while EP2/EP4 signaling can suppress activation in some immune-cell contexts [12,13,19,26,27,28]. The hypothesis therefore does not assume that simultaneous activation of several EP subtypes is inherently analgesic. It predicts a favorable net effect only in a biological state in which immune/stromal inhibitory effects and/or EP3-linked effects outweigh sensory-neuron sensitization; the opposite balance predicts nonresponse or worsening. No direct experiment has yet shown that misoprostol creates such a favorable balance in human neuralgia.

For the present hypothesis, it is therefore preferable not to reduce prostanoid biology to a simple PGE1 ‘anti-inflammatory’ versus PGE2 ‘pro-inflammatory’ dichotomy. PGE2 is a well-established inflammatory and pronociceptive mediator in several settings, yet EP2/EP4 signaling can also restrain activation in selected immune-cell contexts. Misoprostol is a PGE1 analog, but its biological effect is determined by the EP subtypes actually engaged, ligand concentration, exposure pattern, and cellular state. This context dependence provides a mechanistic basis for asking whether low, repeated, and high exposure could produce qualitatively different outcomes. The receptor-specific features relevant to this hypothesis are summarized in Table 1, while the proposed context-dependent balance among receptor, immune-cell, and nociceptor effects is shown schematically in Figure 1.

## 4. A Neuroimmune Pain Unit as the Candidate Substrate

Neuropathic pain emerges from interacting compartments rather than from a single cell type. Peripheral sensory neurons undergo changes in ion-channel expression, membrane excitability, and intracellular signaling; spinal microglia and astrocytes participate in central sensitization; immune cells accumulate at injured nerves; and tissue-resident stromal and vascular cells shape mediator gradients, barrier properties, and repair [3,4,5]. This cellular diversity is central to the hypothesis because the net effect of a nonselective prostanoid agonist may differ across compartments.

### 4.1. Mast Cells and Macrophages

Mast cells are attractive candidates because Shah and Pearce found that misoprostol itself produced a strongly non-monotonic response in isolated human mast cells [25]. Immunologically induced histamine release from human colonic mast cells was inhibited at very low concentrations, with bell-shaped dose–inhibition curves, whereas very high concentrations (≥5 μM) induced histamine release. Human mast cells express several EP subtypes with different effects on activation [26]; EP2 stimulation inhibits human lung mast-cell degranulation [27], and EP2/EP4 signaling suppresses hyperosmolar activation in several human mast-cell preparations [28]. Mast cells also lie close to sensory nerves and vessels and can release histamine, proteases, cytokines, growth factors, and lipid mediators that participate in experimental neuropathic pain [29]. The Shah–Pearce result does not prove that the remembered loss of clinical efficacy at sustained higher doses shares the same mechanism. It does, however, establish in a human inflammatory effector cell that more misoprostol need not produce more inhibition and that sufficiently high exposure can reverse the direction of the response. This finding makes a non-monotonic, concentration- and context-dependent exposure–response relationship a testable component of the present hypothesis rather than a purely clinical speculation.

Microglia provide a central nervous system example of immune cells that can alter synaptic transmission after nerve injury [30]. In the periphery, macrophages are equally plausible contributors: nerve injury recruits and reprograms macrophage populations that communicate with nociceptors, Schwann cells, and vascular elements [31]. A recent human study of painful Morton’s neuroma demonstrated persistent intraneural inflammation, including macrophage-associated molecular signatures that correlated with pain phenotypes [32]. The hypothesis therefore does not require a single ‘misoprostol-sensitive immune cell’. It requires only that transient EP-receptor engagement in one or more interacting compartments can weaken a reinforcing loop.

### 4.2. What Is Meant by a Self-Sustaining Neuroimmune Loop?

Astrocytes deserve explicit consideration in an EP-sensitive network because EP2 stimulation can evoke cAMP-dependent calcium responses in cultured astrocytes [33]. The phrase ‘self-sustaining neuroimmune loop’ is used here as an operational model, not as proof of an autonomous inflammatory circuit. Injured or sensitized sensory fibers can activate immune and stromal cells; their mediators can lower neuronal firing thresholds; sensory fibers can release signals that feedback on surrounding cells; and glia can alter excitatory and inhibitory processing [3,4,5]. These are established elements of neuropathic-pain biology, but their interruption by misoprostol has not been demonstrated.

A prostanoid perturbation can nevertheless outlast the initiating exposure in experimental pain biology. In the study by St-Jacques and Ma, a stabilized PGE2 analog produced nociceptor sensitization/allodynia lasting up to approximately 24 h, associated with increased EP4 synthesis and axonal trafficking [34]. This is indirect and directionally cautionary evidence: the persistent state was pronociceptive, not analgesic, and it still falls short of the recalled 60–72 h interval. No cited experiment demonstrates a 60–72 h analgesic state after misoprostol. Any such duration would require a longer-lived downstream cellular or network alteration and remains a prediction to be tested rather than an explanation already supported.

## 5. Pharmacodynamic Persistence: Distinguishing Receptor, Cellular, and Clinical Time Scales

### 5.1. Definition and Temporal Prediction

In this article, pharmacodynamic persistence means a reproducible pain-relevant functional effect that continues after misoprostol-acid exposure has fallen below measurable or pharmacologically relevant levels. The recurrence-triggered pattern described in Section 1 suggests a time scale to test, not a demonstrated drug effect and not a recommended regimen. It should not be conflated with the historical 600 μg/day regimen used in the prospective neuralgia study [7] or with the approved 800 μg/day gastroprotective regimen (200 μg four times daily) [10]. No evidence-based dose or dosing interval for neuralgic pain can currently be recommended; future dosing must be established by formal pharmacokinetic, pharmacodynamic, safety, and dose-ranging studies.

### 5.2. Candidate Mechanisms and Their Known Time Scales

The proposed mechanisms occupy different time scales and should not be treated as equivalent. EP4 internalization/desensitization is rapid, occurring on a minute’s scale in experimental systems [17,18]. In an unrelated GPCR system, parathyroid-hormone-receptor endocytosis sustained cAMP production for tens of minutes after ligand washout [35], establishing a principle of post-endocytic signaling but not a days-long effect. Contemporary biosensor studies likewise demonstrate endosomal G-protein signaling, but do not show multi-day persistence [20,21,22]. These observations make compartmentalized signaling experimentally plausible while leaving a large temporal gap to 60–72 h.

Longer persistence would therefore have to occur downstream. Transcriptional regulation, receptor synthesis/trafficking, ion-channel phosphorylation or abundance, mediator replenishment, immune-cell phenotype, and multicellular feedback can outlast an initiating receptor pulse. The pain-relevant PGE2-analog model cited above reached approximately 24 h and was pronociceptive [34]. A cellular alteration lasting one day is not evidence for 60–72 h of analgesia, but it identifies a measurable intermediate level. The hypothesis should be evaluated sequentially: persistent receptor signaling (minutes to perhaps hours), persistent cellular alteration (hours to days), persistent change in neuronal excitability or pain behavior, and finally sustained clinical analgesia. Demonstrating an upstream level does not establish any downstream level.

### 5.3. Current Evidence Does Not Support Irreversible EP-Receptor Binding

The hypothesis initially arose because a long symptom-free interval seemed difficult to reconcile with a short-acting drug. Irreversible EP-receptor binding was one intuitive possibility. Current evidence does not support that explanation: misoprostol-acid affinity has been measured under equilibrium binding conditions, the product label describes receptor binding as saturable and reversible, and the EP3 structure shows ordinary non-covalent ligand recognition [10,14,15]. These observations argue against invoking days-long irreversible receptor occupancy, but they do not exclude unmeasured tissue retention, compartment-specific exposure, or an unrecognized active metabolite. Those alternatives require direct analytical pharmacokinetic testing.

### 5.4. Competing Explanations Are Part of the Model

Several non-pharmacological explanations remain at least as plausible as a persistent molecular state. Neuralgic pain is episodic and may vary substantially over hours or days; spontaneous fluctuation and regression to the mean are especially relevant when treatment is started during a severe exacerbation. Expectancy/placebo effects, changes in concomitant treatment or behavior, selective recall of apparent responders, and retrospective timing error can also create an apparently treatment-linked pattern. The recalled 60–72 h interval is particularly vulnerable to circularity because redosing was triggered by pain recurrence: the decision rule itself ensures that recurrence precedes each dose and that any subsequent improvement is temporally associated with dosing. The interval therefore cannot distinguish pharmacodynamic persistence from the natural course of the disorder.

Substantial improvement under blinded placebo conditions further illustrates the problem. In a 2026 randomized, double-blind trial of intravenous fosphenytoin for acute trigeminal neuralgia, 40% of placebo-assigned participants achieved at least 50% improvement in pain intensity at 120 min [36]. That trial does not model multi-day spontaneous remission or the historical misoprostol context, but it shows that marked short-term change can occur without the active intervention. A prospective test must therefore establish baseline attack variability, blind treatment allocation, record attacks continuously, prespecify redosing and rescue criteria, measure exposure and pain on the same time axis, and demonstrate that any post-washout signal exceeds placebo and natural fluctuation. Until then, the 60–72 h recollection is a hypothesis-generating time scale, not evidence of pharmacodynamic persistence. These distinct temporal levels and validation steps are summarized in Figure 2.

## 6. Evidence for and Against the Hypothesis in Pain

### 6.1. Published Neuralgia Signal

The only direct clinical evidence for misoprostol in neuralgia concerns multiple-sclerosis-associated trigeminal neuralgia [6,7,8]. The three reports differ in design, sample size, regimen, outcomes, and major limitations. Together they comprise 28 reported patients, but they do not form an efficacy dataset: the initial report was a small case series, the 2003 study was open and uncontrolled, and the 2012 publication was a three-case letter. No contemporary randomized trial has confirmed the signal, no study linked response to plasma or tissue exposure, and none tested multi-day pharmacodynamic persistence after a single dose.

### 6.2. R.B.B.’s Clinical Experience as the Hypothesis Generator

As detailed once in Section 1, R.B.B.’s own clinical experience generated the question addressed here. It is kept separate from the published evidence and is not used to increase the apparent weight of clinical evidence. The remembered heterogeneity—apparent responses, nonresponses, intolerance, waning benefit, and an apparent tendency for benefit to disappear after several weeks at conventional daily exposure while persisting longer in some lower or intermittently exposed patients—supports only the need for prospective phenotyping and explicit testing of exposure pattern. No response rate, dose optimum, causal inference, or biomarker can be derived from these recollections. Their value is to generate a prespecified prediction: if the clinical impression had a pharmacological basis, concentration–response and repeated-exposure experiments might reveal an intermediate favorable window rather than a monotonic increase in effect with dose.

### 6.3. In Vivo Neural and Vascular Support and Its Limits

In a murine cerebral ischemia model, misoprostol reduced infarct injury, with the reported mechanism consistent with EP2 and/or EP4 rather than EP3 signaling [37]. The result demonstrates that systemically administered misoprostol can influence neural and endothelial biology in vivo. It does not establish analgesia, and the protection occurred without a detectable regional cerebral blood-flow difference. A vascular contribution to pain modulation remains possible, but misoprostol should not be described as a general vasodilator. PGE2 can generate biphasic vascular responses through competing EP pathways [38]; misoprostol altered mucosal blood flow in an experimental gastrointestinal model [39], whereas a controlled human study found no acute improvement in digital circulation after a 400 μg oral dose [40].

These distinctions matter when discussing the eye or cranial pain. Prostaglandin E1, including alprostadil, has vascular, endothelial, antiplatelet, and rheological properties relevant to ischemic retinal disease [41]. Misoprostol is chemically related but differs in receptor engagement, pharmacokinetics, route, and tissue exposure. Misoprostol and PGE1/alprostadil should therefore not be treated as pharmacologically interchangeable. The PGE1 literature supports interest in prostanoid biology; it cannot be transferred directly to misoprostol.

### 6.4. Contrary Prostanoid Pain Evidence

Several findings directly challenge a simple analgesic interpretation and are central to the hypothesis. PGE2 is a well-established pain mediator, and cyclooxygenase inhibition is clinically analgesic in many inflammatory states. Experimentally, PGE2/EP4 signaling can increase EP4 surface availability and sensitize primary sensory neurons [19], PGE2-induced allodynia can depend on EP1 [24], and intravenous PGE2 provokes migraine-like attacks in susceptible patients [42]. Conversely, selective EP3 activation can be antinociceptive in inflamed tissue [23], while EP2/EP4 can suppress activation in some immune-cell contexts [26,27,28]. Misoprostol therefore cannot be expected to relieve pain simply because it is a prostaglandin analog. A favorable response, if real, should be restricted to phenotypes in which the net cellular balance favors inhibitory immune/stromal or EP3-linked effects over nociceptor sensitization. Multiple-sclerosis-associated trigeminal neuralgia is the only phenotype with direct published precedent [6,7,8]; chronic post-injury phenotypes with demonstrable neuroimmune activation are plausible research candidates, whereas migraine and other PGE2-sensitive states are cautionary phenotypes in which exacerbation is biologically plausible. The published clinical evidence is summarized in Table 2.

## 7. Neuropathic Corneal Pain as a Secondary Mechanistic Model

Neuropathic corneal pain (NCP) is clinically distinct from paroxysmal trigeminal neuralgia and has no published analgesic signal for misoprostol. It is retained only as a secondary mechanistic model because corneal afferents belong to the ophthalmic division of the trigeminal system and the peripheral tissue can be examined directly. The TFOS DEWS II report provides a physiological framework [43], while clinical and in vivo confocal microscopy studies show heterogeneous peripheral and central phenotypes, including pain disproportionate to visible surface disease and candidate nerve biomarkers [44,45,46].

The translational advantage is access, not presumed efficacy. Corneal nerves, epithelial cells, immune cells, stromal fibroblasts, tear mediators, and limbal vascular structures can be sampled or imaged in parallel; IVCM and esthesiometry can provide objective readouts. Current management remains heterogeneous [47,48], and contact-lens discomfort can overlap symptomatically without being equivalent to NCP [49]. These features make the ocular surface suitable for receptor-resolved mechanistic experiments before any therapeutic proposal.

Human ocular tissues express EP receptor subtypes [50], and topical misoprostol reduced intraocular pressure in a murine steroid-induced ocular-hypertension model [51]. This establishes local ocular pharmacological activity, not analgesia. Accordingly, topical misoprostol should be considered only a future development route if receptor-dependent antinociceptive effects are first demonstrated in corneal neuron-immune models.

The ocular model is therefore secondary to the systemic neuralgia hypothesis. Its role is to provide experimentally accessible tissue in which exposure, EP-subtype dependence, nerve morphology, inflammatory-cell behavior, and pain-related function can be linked. Failure to demonstrate such a chain would end the ocular branch without weakening or supporting the systemic clinical hypothesis.

Safety precedent also requires caution. Chronic topical prostaglandin analogs used for glaucoma can adversely affect the ocular surface [52], although those agents are mainly FP-receptor drugs rather than EP agonists. Misoprostol is water-sensitive [53], and a topical formulation would require stability, sterility, local-toxicity, retention, and tissue-exposure testing. Nasolacrimal and conjunctival absorption can produce systemic exposure [54]; topical delivery therefore does not remove the reproductive, gastrointestinal, or cardiovascular precautions discussed in Section 9.

Other inflammatory or fibrotic ocular-surface diseases are not proposed as pain indications. Prior observations of altered EP3/EP4 expression [55,56], prostanoid-responsive or disease-associated conjunctival fibroblast behavior [57,58], and inflammatory ocular-surface pathways that affect conjunctival fibroblasts [59] are relevant only as examples of accessible patient-derived systems in which receptor-dependent cellular effects could be tested. The candidate phenotypes and their interpretive value are summarized in Table 3.

## 8. Experimental Strategy: From Receptor Mechanism to Clinical Signal

### 8.1. Receptor-Resolved Concentration–Response Studies

The first experiments should determine whether measured, clinically plausible concentrations of misoprostol acid alter pain-relevant functions. Recombinant human binding data place EP3, EP4, and EP2 affinities in the approximate 8–34 nM range, whereas EP1 affinity is greater than 10 μM [14]. Because the pharmacokinetics of a 100 μg dose in the relevant neural compartment are unknown, in vitro work should not begin with the historical dose as though it defined exposure. A core concentration range spanning the low-nanomolar through low-hundreds-of-nanomolar range should be linked to measured free drug concentration, while higher micromolar conditions are reported separately as supraphysiological mechanistic controls. Crucially, the concentration series should be sufficiently broad and densely sampled to detect bell-shaped, biphasic, or direction-reversing responses rather than assuming monotonicity. Acute low-concentration pulses, sustained exposure, and repeated pulses should be compared because the historical observation concerns both dose and exposure pattern. EP-selective antagonists/agonists, receptor knockdown, or CRISPR perturbation should then resolve subtype contributions.

Expected receptor-specific findings should be prespecified. A favorable effect that is lost after EP3 blockade would support an EP3 contribution; loss after immune-cell EP2 or EP4 disruption would support inhibitory immune signaling; and improvement after neuronal EP2/EP4 blockade would indicate that these receptors were counteracting rather than mediating the favorable response. Pronounced EP1 dependence at low measured concentrations would conflict with the very low EP1 affinity of misoprostol acid [14]. A bell-shaped response, if observed, should be dissected experimentally rather than interpreted phenomenologically: receptor subtype recruitment, desensitization/internalization, changes in receptor surface availability, mediator release, and cell-type-specific responses should be measured across the full concentration range and after repeated exposure. Functional endpoints should include neuronal firing/threshold, calcium or cAMP signals, mediator release, macrophage or mast-cell phenotype, barrier function, and cytotoxicity. The key question is not whether ‘low dose is better’ in the abstract, but whether a reproducible exposure window produces a favorable receptor- and cell-specific state that disappears or reverses outside that window.

### 8.2. Pulse-Washout Experiments for Persistence

Pulse-washout experiments should treat persistent receptor signaling, persistent cellular alteration, and persistent pain-related function as separate endpoints. After a brief exposure and verified drug removal, receptor localization and compartmentalized cAMP/G-protein activity should be measured first. Transcription, mediator recovery, receptor/ion-channel abundance, and immune-cell phenotype should then be followed on longer time scales; only thereafter should neuronal excitability or pain behavior be assessed. Antagonists applied during the pulse, after washout, or during a later phase can distinguish initiation from ongoing receptor dependence. Failure at any level is a go/no-go stop rather than a reason to infer the next level from an upstream signal.

General GPCR biology justifies measuring subcellular signaling but does not define its duration in this system [20,21,22,35]. The available precedents span minutes for post-endocytic cAMP signaling [35] and up to roughly 24 h for a PGE2-analog-associated nociceptor state [34]; neither demonstrates 60–72 h misoprostol analgesia. Experiments should therefore report the actual decay constant or time to baseline for each molecular and functional readout rather than using the generic term ‘sustained’.

### 8.3. Pharmacokinetic–Pharmacodynamic Coupling

In animal and human studies, plasma misoprostol acid should be sampled densely around dosing while tissue concentrations are measured whenever feasible. The key comparison is between free exposure and the receptor-affinity/functional-response range, not plasma half-life alone. Target-compartment PK is essential because a short plasma half-life does not exclude slower tissue elimination, local partitioning, or metabolite formation. Analytical methods should therefore quantify parent misoprostol where relevant, misoprostol acid, and identifiable metabolites in plasma and target tissue, while pain-related readouts continue after systemic clearance.

### 8.4. Adult Clinical Signal Finding

Clinical testing should follow, not precede, reproducible receptor-dependent and pain-relevant effects at plausible exposure. Multiple-sclerosis-associated trigeminal neuralgia is the logical first human phenotype because it alone has a published misoprostol signal [6,7,8]. No dose is recommended by the present article. A dose-ranging stage should establish exposure, tolerability, receptor-informed biological plausibility, and stopping rules before any efficacy-oriented comparison. Because both the historical recollection and the mast-cell data [25] raise the possibility of non-monotonicity, dose-ranging should not be designed solely to identify a maximum tolerated or progressively more active dose. It should be capable of identifying an intermediate exposure window, loss of response during repeated conventional exposure, and any reversal toward pronociceptive or pro-inflammatory effects at higher exposure.

To avoid the circularity of recurrence-triggered historical dosing, the primary clinical design should make time-to-recurrence independent of the patient’s knowledge of treatment. Options include fixed randomized active/placebo dosing with continuous electronic attack recording, or a blinded event-driven design in which identical active/placebo doses are assigned by a prespecified randomization rule after a predefined recurrence threshold. Baseline run-in, rescue medication, washout, adherence verification, and prespecified redosing criteria are essential. Pregnancy-related eligibility, cardiovascular assessment, gastrointestinal monitoring, and systemic exposure measurement must be built into the protocol from the outset.

The staged program in Table 4 now separates three distinct persistence claims: continued receptor signaling after washout, a longer-lived cellular alteration after receptor signaling has ended, and a sustained pain-related functional/clinical effect. Each level has an independent go/no-go criterion. A positive molecular result cannot be used as a surrogate for analgesia, and a clinical signal without exposure and mechanistic plausibility would remain uninterpretable.

## 9. Safety, Developmental Boundaries, and Risks of Overgeneralization

Misoprostol has major reproductive risks, including uterine contractions, pregnancy loss, premature birth, fetal harm, and uterine rupture [10]. Gastrointestinal adverse effects, particularly diarrhea and abdominal cramping, can limit exposure, and the prescribing information advises caution in patients with pre-existing cardiovascular disease [10]. Any future clinical investigation must therefore include explicit reproductive eligibility criteria, confirmation that participants who could become pregnant are not pregnant at enrollment and during treatment as appropriate, effective contraception consistent with the label, predefined gastrointestinal monitoring, cardiovascular history/risk assessment, adverse-event stopping rules, and measurement of systemic exposure. These precautions apply even to topical ocular development because conjunctival and nasolacrimal absorption can be systemic [54].

Developmental age deserves separate caution. Current United States prescribing information states that safety and effectiveness in pediatric patients have not been established [10]. Synaptic refinement continues through adolescence and early adulthood in humans [60], and microglia participate in adolescent synapse elimination in experimental models [61]. No evidence shows that misoprostol disrupts synaptic pruning in children or adolescents. Nevertheless, because the hypothesis explicitly invokes neuroimmune signaling and possible post-exposure cellular persistence, initial clinical investigation should be restricted to adults.

Clinical feasibility also depends on access. Availability of misoprostol-only products varies substantially among countries and has changed over time; a 2026 analysis of 66 countries documented marked regional differences and declining misoprostol-only or misoprostol-NSAID sales in several markets [62]. A trial site would therefore need to confirm lawful prescribing, pharmacy supply, formulation, storage, and reproductive-risk controls before enrollment. If misoprostol itself proves impractical or pharmacologically unsuitable, the mechanistic program remains informative: once a receptor/cell compartment is identified, development could move to a more selective EP ligand rather than forcing clinical development of misoprostol.

The same restraint should apply to broader disease claims. R.B.B. recalls that the clinical observations that originally drew his attention to misoprostol were not confined to one neuralgic phenotype and included other neurological and non-neurological conditions in which chronic inflammatory or neuroimmune mechanisms might be relevant. Because these additional observations are undocumented and often involved isolated individuals, they are not presented here as evidence for additional indications. Cerebral ischemia shows that misoprostol can influence neural tissue in vivo [37], but it does not justify extrapolation to Alzheimer’s disease, Parkinson’s disease, epilepsy, chronic traumatic encephalopathy, psychiatric disorders, systemic autoimmune disease, or other chronic inflammatory disorders. In several of those fields, the desired direction of EP signaling may differ by disease stage and cell type. The broader recollections therefore support only a future question: whether a non-monotonic, exposure-dependent prostanoid effect might be relevant to selected chronic neuroimmune or inflammatory states. The present article remains a molecular pain hypothesis with one accessible ocular extension, not a general repurposing manifesto.

## 10. Limitations and Criteria for Rejection of the Hypothesis

The strongest limitation is the evidentiary gap between the hypothesis generator and the proposed mechanism. The published clinical signal is sparse, old, and uncontrolled; R.B.B.’s broader physician recollections—including the apparent contrast between waning benefit during conventional daily exposure and longer perceived benefit with lower or intermittent exposure—are unverifiable and are not clinical evidence. No direct study has shown that misoprostol causes post-washout EP signaling, a persistent cellular alteration, sustained analgesia in neural tissue, or a clinically meaningful bell-shaped analgesic dose–response. Plasma clearance does not define tissue clearance, clinically relevant receptor occupancy is unknown, and a nonselective EP ligand may be pronociceptive in some phenotypes. The ocular branch is secondary and similarly lacks a direct analgesic signal.

The hypothesis should therefore be rejected or substantially revised if measured clinically plausible exposure does not engage a relevant EP pathway; if receptor-proximal signaling does not persist after verified washout; if a persistent cellular alteration is absent after receptor signaling subsides; if such a cellular alteration fails to improve neuronal excitability or pain behavior; if selective EP manipulation does not reproduce or block the relevant effect; if activity requires supraphysiological or cytotoxic concentrations; if repeated exposure produces sensitization; or if blinded adult studies show no analgesic/time-to-recurrence signal beyond placebo and natural fluctuation. These are independent failure points: success at one level does not rescue failure at the next.

## 11. Conclusions

The present hypothesis originates from a clinical puzzle encountered by R.B.B. during decades of adult neurological practice, but it is not an attempt to convert recollection into evidence. Published misoprostol data in multiple-sclerosis-associated trigeminal neuralgia provide only a small independent precedent. Current evidence supports rapid conversion to misoprostol acid, reversible EP-receptor binding, context-dependent EP biology, and at least one human mast-cell response in which inhibition is bell-shaped and reverses at very high concentrations [25]; it does not demonstrate multi-day post-washout EP signaling or analgesia. The remembered tendency for benefit to wane during sustained conventional exposure while appearing more durable with lower or intermittent exposure therefore becomes a testable prediction, not a therapeutic claim. The productive questions are sequential and exposure-aware: does clinically plausible exposure engage a relevant EP subtype; is there a reproducible favorable exposure window rather than a monotonic dose–response; does receptor-proximal signaling outlast washout; does a longer-lived cellular change follow; does neuronal excitability improve rather than worsen; and does blinded clinical analgesia persist independently of dosing decisions? Failure at any step should stop or redirect the program. In this form, R.B.B.’s experience remains the origin of the hypothesis while the evidentiary burden is placed entirely on prospective molecular and clinical testing.

## Figures and Tables

**Figure 1:**
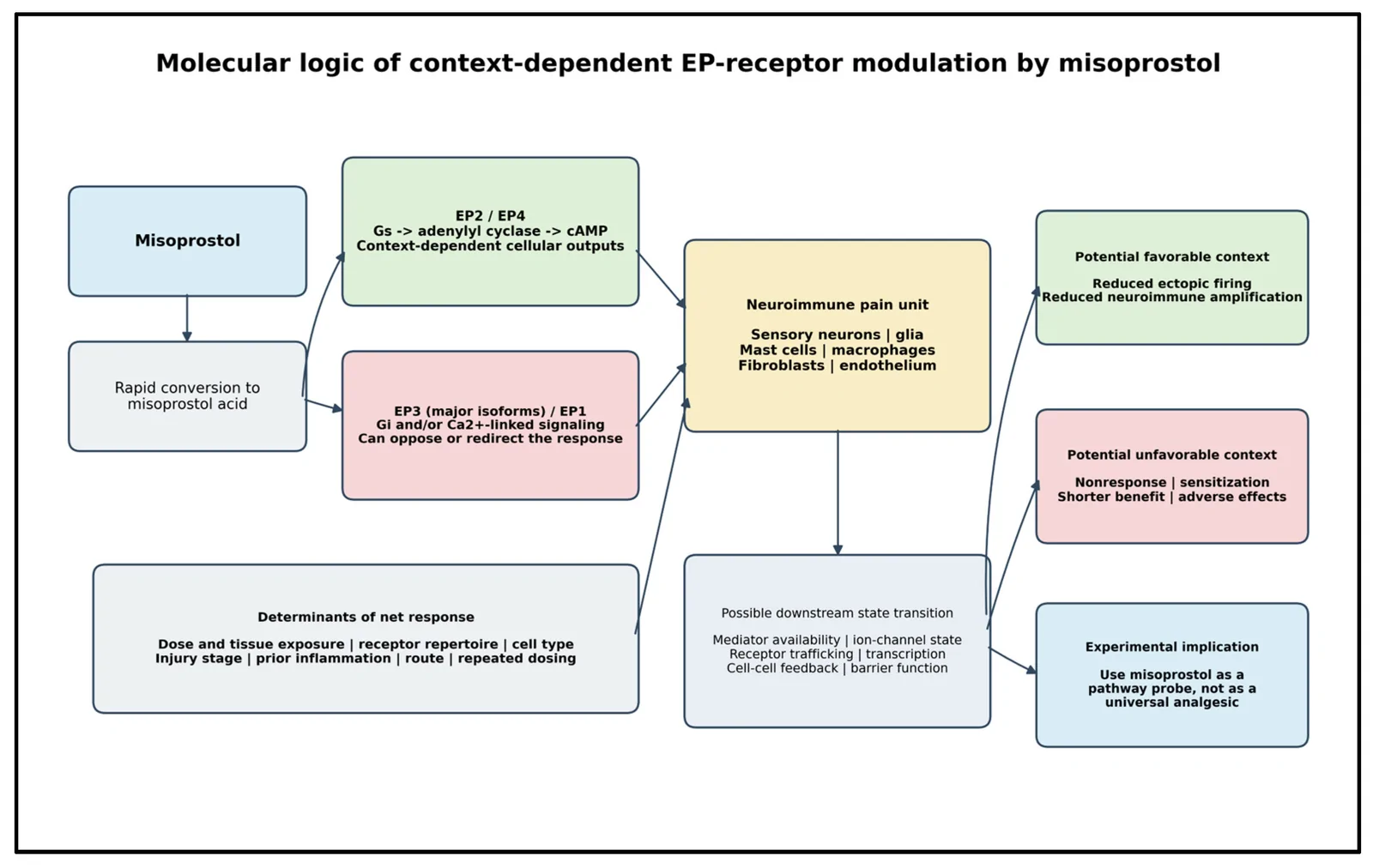
Molecular logic of context-dependent EP-receptor modulation by misoprostol. Rapid conversion to misoprostol acid and direct misoprostol receptor-binding/structural evidence are supported by [9,10,11,14,15]; general EP-receptor pharmacology and EP4 structural context are provided by [12,13,16]. The downstream receptor-regulation, immune-cell, and nociceptor arrows synthesize mainly indirect evidence from PGE2, selective prostanoid ligands, or broader GPCR biology [17,18,19,20,21,22,23,24,26,27,28] and should be interpreted as hypotheses rather than demonstrated consequences of misoprostol in neuralgia. The figure therefore depicts possible directions of effect, not established causality or relative effect size. In the schematic, blue/gray boxes denote exposure, determinants, downstream-state, or experimental elements; green and red boxes denote potentially favorable and unfavorable pathways/outcomes, respectively; yellow denotes the integrated neuroimmune pain unit. Arrows indicate proposed directions of influence, not established causal links.

**Figure 2:**
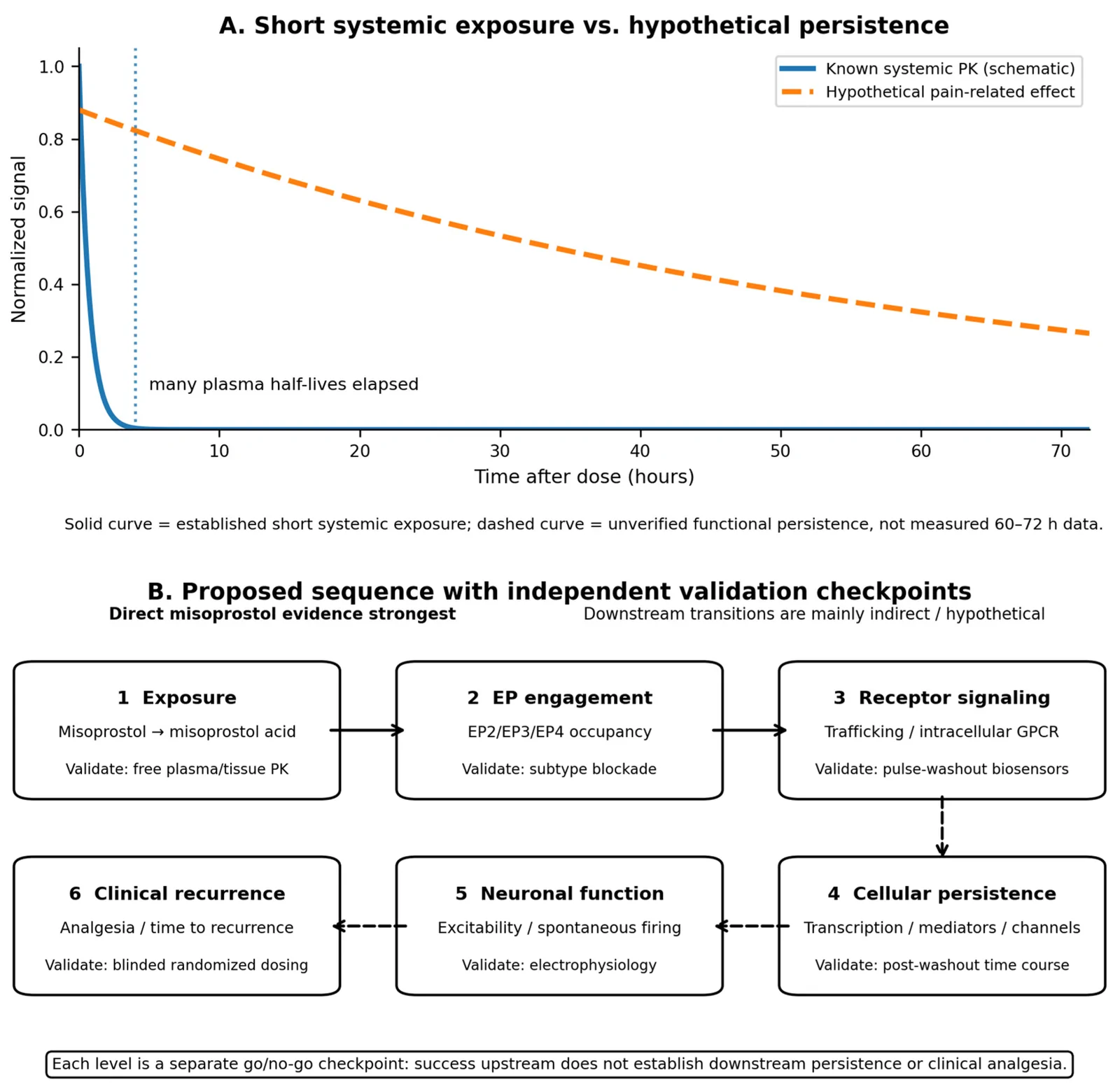
Separating pharmacokinetic exposure, mechanistic persistence, and clinical analgesia. Panel (**A**) distinguishes the established short systemic exposure of misoprostol acid (solid schematic curve) from a hypothetical pain-related effect (dashed curve). The dashed curve is conceptual and is not a measured 60–72 h response. Panel (**B**) separates six validation levels: exposure, EP-receptor engagement, receptor trafficking/compartmentalized signaling, persistent cellular alteration, altered neuronal excitability, and clinical analgesia/time to recurrence. Exposure and direct misoprostol receptor-binding/structural evidence is supported by [9,10,11,14,15]. Receptor regulation and downstream persistence hypotheses rely mainly on general EP-receptor pharmacology, PGE2/other prostanoid data, or broader GPCR evidence [12,13,16,17,18,19,20,21,22,23,24,26,27,28,29,30,31,32,33,34,35], with direct misoprostol cellular evidence limited to the context-dependent mast-cell effects in [25]. Each level must be tested independently; a positive result at one level does not establish the next.

**Table 1:** Molecular features of EP receptors relevant to the misoprostol hypothesis.

Receptor	Canonical Coupling/Misoprostol-Acid Affinity	Direct Pain-Related Evidence	Why the Net Effect Is Uncertain	Decisive Receptor-Specific Test
EP1	Primarily Gq/Ca^2+^; very low misoprostol-acid affinity (>10 μM) [14]	PGE2-induced allodynia was abolished in EP1-deficient mice [24].	EP1 is pronociceptive in that model, but low misoprostol-acid affinity makes major EP1 occupancy at low exposure uncertain.	If EP1 blockade changes the effect only at supraphysiological exposure, EP1 is unlikely to explain a low-exposure–response; strong EP1 dependence at measured low tissue concentrations would challenge the current affinity-based model.
EP2	Gs → cAMP; Ki ~ 34 nM [14]	EP2 can suppress mast-cell activation [27,28], whereas neuronal consequences are context-dependent [12,13].	An immune-cell benefit could coexist with neuronal cAMP-dependent sensitization.	Block/knock down EP2 during a misoprostol-acid pulse. Loss of a favorable immune/functional effect would support EP2 involvement; improvement after EP2 blockade would indicate a net pronociceptive EP2 contribution.
EP3	Multiple isoforms; Gi common; Ki ~ 7.9 nM [14]	Selective EP3 activation was antinociceptive in inflamed tissue [23], but PGE2 allodynia was preserved in EP3-deficient mice [24].	Isoform, cell type, and disease context can reverse interpretation; EP3 is not a universally analgesic receptor.	Test selective EP3 agonism and EP3 blockade/knockdown. Reproduction and blockade of the same post-washout functional phenotype would support EP3 dependence.
EP4	Gs → cAMP; Ki ~ 23 nM [14]	EP4 undergoes rapid internalization [17,18]; PGE2 can increase sensory-neuron EP4 surface availability and sensitization [19], while EP4 can restrain mast-cell activation [26,28].	The same receptor can be favorable in immune cells and unfavorable in nociceptors.	Resolve cell type explicitly. A favorable effect that disappears with immune-cell EP4 disruption but is enhanced by neuronal EP4 blockade would support a compartment-dependent mechanism.

Abbreviations: EP, E-type prostanoid receptor; cAMP, cyclic adenosine monophosphate; Gq, Gq protein; Gi, inhibitory G protein; Gs, stimulatory G protein.

**Table 2:** Structured assessment of the published clinical evidence for misoprostol in multiple-sclerosis-associated trigeminal neuralgia.

Study/Design/Population	N	Dose and Duration	Reported Outcomes and Major Limitations
Reder & Arnason, 1995 [6] Case series; refractory MS-associated TN	7	Case-based oral misoprostol; detailed dose and treatment duration are not available in the PubMed abstract.	Pain relief reported in 6/7. No control or blinding; very small sample; no standardized modern outcomes, exposure measurements, or randomized confirmation.
DMKG Study Group, 2003 [7] Open prospective study; refractory MS-associated TN	18 completed	600 μg/day. Improvement was reported beginning about day 5; total study duration is not specified in the PubMed abstract.	>50% reduction in attack frequency and pain intensity in 14/18. Mild transient adverse effects in 3; one patient stopped after the study because of severe menorrhagia. No placebo, blinding, or PK-PD assessment.
Pfau et al., 2012 [8] Letter/case series; therapy-resistant MS-associated TN	3	Case-based oral treatment; 3 × 200 μg/day is explicitly described for the first case. Follow-up and concomitant medication changes were not standardized as a trial.	Rapid cessation of attacks was reported in the case descriptions. Case-report design, selection/reporting bias, concomitant treatment changes, and no control group prevent efficacy estimation.

**Table 3:** Candidate pain phenotypes and their interpretive value.

Phenotype	Why Informative	Main Uncertainty	First Decisive Test
MS-associated trigeminal neuralgia	Only pain phenotype with a published misoprostol signal [6,7,8].	Responder biology and optimal exposure are unknown.	Adult randomized crossover or randomized N-of-1 series after receptor- and exposure-informed dose selection.
Classical trigeminal or glossopharyngeal neuralgia	Focal paroxysmal pain with possible neurovascular, glial, and immune components.	Diagnosis or compression alone may not identify an EP-sensitive state.	Phenotype-rich blinded pilot with attack counts, washout, rescue therapy, sensory profiling, and timed pharmacokinetics.
Postherpetic neuralgia	Peripheral nerve injury and chronic neuroimmune sensitization provide a distinct mechanism.	Acute zoster-associated pain and established postherpetic neuralgia should not be pooled; disease duration and sensory phenotype may determine response.	Stratified adult pilot with quantitative sensory testing and continuous time-to-recurrence recording.
Neuropathic corneal pain—secondary mechanistic model	Accessible trigeminal afferents and ocular-surface cells; IVCM/esthesiometry can link tissue exposure to nerve and immune readouts [43,44,45,46,47,48,49,50,51].	No direct misoprostol analgesic evidence; clinical phenotype differs from paroxysmal TN; local and systemic safety remain unproven.	Corneal neuron-immune model with EP2/EP3/EP4 perturbation and measured exposure; only if favorable, proceed to topical PK/safety and blinded exploratory testing.
Migraine—cautionary phenotype	Provides a direct test of the possibility that prostanoid activation facilitates rather than suppresses trigeminal pain.	PGE2 can provoke migraine-like attacks and EP4 can sensitize nociceptors [19,42]; symptom exacerbation is biologically plausible.	Use receptor-resolved trigeminal/meningeal models as a safety counter-screen; do not pursue therapeutic exposure if pronociceptive signaling dominates.

**Table 4:** Staged program separating exposure, receptor signaling, cellular persistence, pain-related function, safety/feasibility, and clinical analgesia.

Stage	Independent Question	Approach and Go/No-Go Criterion
1. Exposure and receptor engagement	Does clinically plausible free misoprostol acid reach and engage relevant EP receptors?	Measure free plasma/tissue exposure; bracket the EP2/EP3/EP4 affinity range with a sufficiently dense concentration series to detect monotonic, bell-shaped, or biphasic responses; compare acute, sustained, and repeated exposure; use receptor-selective perturbation. Go only if effects occur at non-cytotoxic, plausibly achievable exposure, show reproducible subtype dependence, and can be distinguished from high-concentration reversal or toxicity.
2. Persistent receptor signaling	Does receptor-proximal signaling continue after verified washout?	Pulse-washout cAMP/G-protein biosensors, receptor localization, delayed antagonism. Go only if post-washout signaling is reproducible and exceeds assay/drug-removal artifacts; record its actual duration.
3. Persistent cellular alteration	Does a cellular state outlast receptor-proximal signaling?	Serial transcription, receptor/ion-channel abundance, mediator recovery, immune-cell phenotype. Go only if a reproducible downstream state persists after receptor-proximal signaling has returned toward baseline.
4. Pain-relevant functional effect	Does the cellular alteration favorably change neuronal excitability or pain behavior?	Electrophysiology, evoked/spontaneous firing, neuron-immune co-culture, disease-relevant pain models. Go only if function improves; pronociception or sensitization is a stop/safety signal.
5. PK, safety, formulation, feasibility	Can the target compartment be exposed safely and can the drug be obtained/regulatorily used?	Systemic/target-tissue PK; reproductive, GI, cardiovascular and ocular safety; formulation where relevant; site-specific pharmacy/regulatory feasibility. Stop or shift to receptor-selective ligands if exposure or access is unsuitable.
6. Blinded clinical signal	Is analgesia reproducible independently of recurrence-triggered dosing?	Adult randomized crossover/N-of-1/small parallel study with fixed or blinded randomized dosing, electronic time-to-recurrence, rescue therapy, PK, exposure-pattern/dose-ranging, and prespecified safety rules. Designs should be capable of detecting an intermediate favorable exposure window as well as loss or reversal of benefit with repeated/higher exposure. Clinical benefit must be demonstrated independently of molecular persistence.

## Data Availability

No new datasets were created or analyzed in this study. Data sharing is not applicable to this article.
